# Polyethylene Glycol Exposure with Antihemophilic Factor (Recombinant), PEGylated (rurioctocog alfa pegol) and Other Therapies Indicated for the Pediatric Population: History and Safety

**DOI:** 10.3390/ph11030075

**Published:** 2018-07-26

**Authors:** Reinhard Stidl, Michael Denne, Jimena Goldstine, Bill Kadish, Katherine I. Korakas, Peter L. Turecek

**Affiliations:** 1 Baxalta Innovations GmbH, Shire, Industriestraße 67, 1220 Vienna, Austria; reinhard.stidl@shire.com; 2 Shire, Bank of America Plaza, 540 W Madison St, Chicago, IL 60661, USA; michael.denne@shire.com (M.D.); jimenag@gmail.com (J.G.); 3 PAREXEL International, 433 Hackensack Avenue, 10th Floor, Hackensack, NJ 07601, USA; Bill.Kadish@ppsigroup.com (B.K.); Kate.Korakas@PAREXEL.com (K.I.K.)

**Keywords:** PEGylation, safety, pediatric, biologics, polyethylene glycol, excipient, conjugation, polysorbate, hemophilia, rurioctocog alfa pegol

## Abstract

Polyethylene glycol (PEG) is an inert, water soluble polymer, used for decades in pharmaceuticals. Although PEG is considered safe, concerns persist about the potential adverse effects of long-term exposure to PEG-containing therapies, specifically in children, following the introduction of PEGylated recombinant factor products used for the treatment of hemophilia. Given the absence of long-term surveillance data, and to evaluate the potential risk, we estimated PEG exposure in the pediatric population receiving PEGylated therapies with pediatric indications administered intravenously or intramuscularly. We used a range of pediatric weights and doses based on prescribing information (PI) or treatment guidelines. PIs and reporting websites were searched for information about adverse events (AEs). For a child weighing 50 kg on the highest prophylactic dose of a FVIII product, the range of total PEG exposure was 40–21,840 mg/year; for factor IX (FIX) products, the range was 13–1342 mg/year; and for other products, the range was 383–26,743 mg/year, primarily as a derivative excipient. No AE patterns attributable to PEG were found for any of these products, including potential renal, neurological, or hepatic AEs. Our analyses suggest the pediatric population has had substantial exposure to PEG for several decades, with no evidence of adverse consequences.

## 1. Introduction

Polyethylene glycol (PEG) is a chemically inert, amphiphilic polymer of ethylene diol units that can be manufactured in various sizes and molecular weights. PEG has been used as an excipient in many pharmaceuticals, cosmetics, and consumer products for decades, and as a conjugate with biologicals (i.e., PEGylation) since 1990 [1,2,3,4,5]. Currently, there are 20 PEGylated therapies with diverse indications in clinical use in the United States and Europe [1,6,7,8].

PEGylation technology involves the covalent attachment of a PEG molecule to a peptide, protein, small molecule or nucleic acid to prolong the therapy’s half-life in circulation, to decrease plasma clearance, and/or to alter biodistribution, in comparison with non-PEGylated versions, thereby decreasing the dosing frequency required to maintain a therapeutic level [9]. PEGylation of biotherapeutics has been applied in many clinical disorders, including hemophilia A and B [1,7,10,11]. For example, with respect to factor VIII (FVIII) replacement therapy in hemophilia A, Antihemophilic Factor (Recombinant), PEGylated (rurioctocog alfa pegol, BAX 855, SHP660; ADYNOVI^®^/ADYNOVATE^®^, Shire, Lexington, MA, USA) has a half-life that is 1.4–1.5 fold longer [6,11] than Antihemophilic Factor (Recombinant) (ADVATE^®^, Shire Lexington, MA, USA), allowing for two doses per week rather than three for prophylaxis [6,11,12].

There have been concerns about PEG safety in pediatric hemophilia patients based on the amount of PEG exposure from lifelong prophylaxis beginning at a very early age [13,14]. The standard of care for a hemophilia A patient has changed from on-demand treatment, defined as infusing factor concentrate in response to a bleed, to prophylactic treatment, in which patients receive regular replacement of FVIII to maintain a minimum effective FVIII level [15]. Now that prophylaxis is routinely used, the exposure to factor replacement therapy typically begins at a very early age (often less than 1 year old) and is continuous throughout childhood and adolescence.

During preclinical development, PEGylated therapeutics, like other pharmaceuticals, undergo toxicological evaluation in animal models, where supra-therapeutic doses are repeatedly administered [3,4]. The major cause for concern about PEG stems from animal model toxicological studies where repetitive dosing at very high levels led to vacuolation in particular cells [14]. In a 2013 published industry survey, vacuolation was observed in toxicology studies for five of the 11 approved PEGylated therapies [16]. Vacuolation is considered a normal physiological process by which the cell tries to remove foreign entities [17]. In animal cells, vacuoles can be formed by different mechanisms in different cellular compartments (e.g., endosomes, lysosomes, endothelial reticulum), but are usually non-existent or small in comparison to those in plant and fungi cells, but can grow in size spontaneously when exposed to bacteria, viral pathogens, and various naturally occurring or artificial small molecule compounds [17,18,19,20]. The vacuolation phenomenon seen in mammalian cell cultures can be transient or irreversible. Vacuolation is reversible if the effect of the vacuolation-inducing agent on the cell cycle and cell migration ceases upon removal of the agent [17]. In contrast, irreversible vacuolation is associated with cytopathological circumstances leading to cell death, as long as the cytotoxic inducer is present. In some studies, vacuolation was followed by cell death, but it was determined that vacuolation did not directly cause cell death but was possibly a side effect, and vacuolation may more commonly improve cell survival by decreasing stress [17]. Despite the extensive research on cytotoxic inducers, the mechanism of vacuolation, and downstream effects remain unclear, and no clear functional impact of vacuolation has been reported [2,16].

Several preclinical toxicology studies on PEGylated therapies have led to an observation of vacuolation propensity in renal tubule cells, macrophages and epithelial cells of the choroid plexus [10]. In one study, it was shown that exposure to high doses of PEGylated tumor necrosis factor (TNF) protein over a period of three months led to vacuolation of renal cortical tubular epithelium cells in Sprague-Dawley rats. After a 2-month recovery period, the vacuolation diminished and was not associated with pathological alterations [21]. A potentially more serious concern is the observation of vacuolation in epithelial cells of the choroid plexus, which are the main source of cerebrospinal fluid and are a central component of the blood-cerebrospinal fluid barrier. Recently, Rudmann et al. reported a correlation between the molecular weight of unconjugated linear PEG and vacuolation in rats. The authors compared the effects of repeated injections of 100 mg kg^−1^ of 10 kDa PEG administered daily, 20 kDa PEG administered on alternate days, and 40 kDa PEG administered weekly, all of which constitute very high doses of PEG. It was observed that in rats given the highest molecular weight PEG, immune-historeactivity to PEG decreased in renal tubule cells, but increased in splenic macrophages and choroid plexus epithelial cells. Vacuolation was only observed in macrophages, choroid plexus epithelial cells and renal tubular epithelial cells [22] treated with 40 kDa PEG. Due to these preclinical observations, when we analyzed PEG-containing therapies for clinical manifestations, we looked for hepatic, renal, and neurological symptoms.

Small excipient PEGs are added to products due to their low toxicity, favorable solubility, and low viscosity [4]. Although it is recognized that many consumer and pharmaceutical products contain PEG, there may be a lack of awareness regarding the use of large quantities of excipient PEG in FVIII and factor IX (FIX) therapies that have been approved decades ago for hemophilia A and B, respectively, as well as in other therapies administered long term in the pediatric population. In fact, excipient PEG has been present as PEG 3350 in plasma-derived FVIII products since 1966 (the number listed after PEG is the molecular weight in Daltons (Da)) [6,23].

To date, there is no evidence that PEG, whether excipient, derivative, or conjugate, is the cause of any adverse event (AE) associated with PEG-containing therapies administered at FDA-approved doses in humans [13,14,16]. Nor has any PEG-specific risk in humans been identified, regardless of PEG size or underlying medical condition [2,14]. Unfortunately, for PEGylated therapies generally, and rurioctocog alfa pegol specifically, there are no long-term surveillance data in the pediatric population. Because of this data gap, concerns about potential toxic effects of long-term exposure to PEG-containing therapies persist.

Given the lack of long-term surveillance data of PEG exposures in children, we developed an alternative approach to help healthcare providers (HCPs) understand the magnitude of exposure to PEG and its potential toxicity for rurioctocog alfa pegol, and all other FDA-approved, pediatric-indicated parental therapies with conjugated, derivative, and/or excipient PEG. We estimated the cumulative exposure over one year, based on the calculation of the amount of PEG in a therapeutic dose for pediatric patients of different weights. To evaluate potential PEG-related toxicity, we investigated all publicly available AEs for PEG-containing pediatric-indicated therapies with intravenous (IV) or intramuscular (IM) routes of administration to determine if there were any general trends or any AEs that might be related to vacuolation [13,14] in the central nervous system (CNS), kidney, or liver. To address this concern, we determined the total PEG content of rurioctocog alfa pegol and all other FDA-approved, pediatric-indicated parental therapies with conjugated, derivative, and/or excipient PEG. We calculated total exposure and compared them in terms of total exposure and side effects. Our objective was to estimate annual PEG exposure from each product as commonly prescribed, and to determine whether any publicly reported AEs could be attributed specifically to PEG.

## 2. Materials and Methods

Oral and subcutaneous therapies were not included so that a direct comparison could be carried out between IV or IM therapies, without confounding the analyses with different biodistribution, pharmacokinetics, and pharmacodynamics. FDA-approved therapies with pediatric indications were analyzed for PEG exposure per dose and per year. For comparative purposes, we grouped FVIII products together as primary comparators to rurioctocog alfa pegol. We also grouped FIX products together and PEG-containing therapies indicated in the pediatric population for various other diseases (e.g., primary immunodeficiency, leukemia, and renal cell carcinoma). Our analysis was not based on real-world case studies, but on theoretical weights of children: a 10 kg 1-year-old and a 50 kg 14-year-old [24].

Several therapies with PEG were not included in the analysis due to inadequate information to perform the calculations. For PEGylated therapies, in order to calculate PEG amounts in a dose and per year, it was necessary to obtain information on the pediatric therapeutic dose, conjugate PEG molecular weight, total molecular weight of the biological, and the specific activity. For therapies with excipient or derivative PEG, it was necessary to have information on the pediatric dose, concentration of PEG per vial, and the ratio of PEG molecular weight to derivative molecular weight. We were unable to locate the amount of polysorbate 20 in antihemophilic factor (recombinant) or Fc fusion protein (Eloctate^®^, Bioverativ Therapeutics Inc., Waltham, MA, USA), and thus exposure calculations were not performed. Other medications for which we were unable to calculate exposures include: Immune Globulin Injection (Human), 10% Caprylate/Chromatography Purified (Gamunex-C^®^, Grifols Therapeutics Inc., Research Triangle Park, NC, USA), Immune Globulin Intravenous (Human), 10% Liquid (Privigen^®^, CSL Behring LLC, Kankakee, IL, USA), Immune Globulin Intravenous (Human) (Carimune^®^, CSL Behring LLC, Kankakee, IL, USA), Immune Globulin Injection (Human) 10% Caprylate/Chromatography Purified (Gammaked^®^, Grifols Therapeutics Inc., Research Triangle Park, NC, USA) and amiodarone hydrochloride (Cordarone X^®^, Sanofi-Aventis Australia, Macquarie Park, NSW, Australia).

If we had only looked at PEGylated biologicals with pediatric indications, we would be limited to pegaspargase (ONCASPAR^®^, Shire, Lexington, MA, USA) and nonacog beta pegol (Rebinyn^®^/Refixia^®^, Novo Nordisk A/S, Bagsvaerd, Denmark). In order to increase the breadth of our analysis, we included all pediatric-indicated therapies with excipient and derivative PEG administered via IV or IM routes. Polysorbate 80 (PS80), polysorbate 20 (PS20), and poloxamer 188 (P188) are excipients widely used in consumer products and pharmaceuticals. Since PEG is a part of the chemical structure of these excipients and is found to be released as a PEG derivative [25,26], we included therapies that contained these additives. We differentiated the types of PEG as conjugate (i.e., PEGylated therapies), excipient (i.e., PEG 3350), and derivative (i.e., PS80). Therapies with excipient and derivative PEG were found by searching FDA.gov and Google™ for therapies with “PEG”, “polyethylene glycol”, “polysorbate”, “polysorbate 20”, “polysorbate 80”, “poloxamer”, “poloxamer 188”, “PS 80”, and “PS 20”.

**Figure 1:**
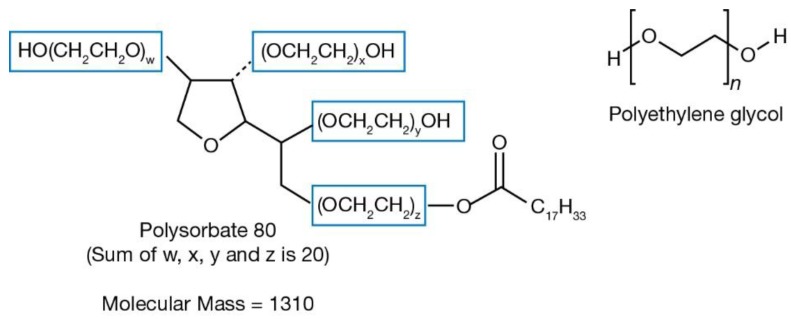
Chemical structures of Polysorbate 80 and polyethylene glycol (PEG). PEG has a chemical formula of H−(O−CH_2_−CH_2_)_n_−OH. The PEG moieties are highlighted in blue boxes within the polysorbate 80 molecule.

To quantify excipient and derivative PEG, we included free excipient PEG of various molecular weights and derivatives with chemical structures containing PEG chains (PS20, PS80, and P188). The proportion of PEG within the chemical structure was calculated. For example, PS80 (MW 1310 g/mol) [27] is a combined molecule of three parts: oleic acid (MW 282.47 g/mol), sorbitol (MW 182.17 g/mol), and PEG (1310 − 282.47 − 182.17 = 845.36 g/mol) (Figure 1). Therefore, PEG comprised 64.5% of PS80.

For FVIII and FIX therapies, PEG exposure was calculated based on a range of prophylaxis dosages recommended in the 2013 World Federation of Hemophilia Guidelines (25–40 IU/kg) [28]. For extended half-life recombinant (r) FVIII and rFIX products and all other products, exposure was calculated based on prescribing information (PI), including a given dosage range.

For all PEG-containing therapies, two weights were used to cover the theoretical pediatric population range: 10 and 50 kg, with exceptions pertaining to therapies that are indicated for a specific population age within the PI. The formula used to calculate PEG exposure per dose was:

$$\begin{array}{l} PEGylated\;Biologic:PEG\;(mg)=Patient\;weight\;(kg)\times Dose\;(IU\;/kg)\times (PEG\;MW/Total\;MW)/Specific\;Activity\\ Excipient\;or\;derivative\;PEG\;(mg)\;=\;Patient\;weight\;(kg)\times Dose\;(IU\;/kg)\times PEG\;concentration\;(mg/IU)\times PEG\;ratio* \end{array}$$

* Ratio: For free PEG, PEG/PEG = 1; for PS80, PEG/PS80 = 0.645; for PS20, PEG/PS20 = 0.84; for P188, PEG/P188 = 0.69.

Many of the products in this evaluation are available in multiple dosage vial sizes, and for some therapies, the excipient and/or derivative PEG weight amount was the same weight amount per vial (i.e., 0.1 mg PS80 in the 250, 500, 1000 IU dosage vials), while others had different amounts of PEG per dosage vial (i.e., 0.1 mg PS80 in the 250 and 500 IU dosage vials, 0.2 mg PS80 in the 1000 IU dosage vial). Instead of choosing one dosage vial for the hypothetical 10 kg and the 50 kg patient, for this analysis, vial sizes were pragmatically chosen to minimize factor waste leading to final PEG amounts that did not necessarily trend with increasing dosage amounts. Also, in the cases of doses that require a combination of two vials, the dosage vials were again chosen to minimize waste, which is a common practice amongst prescribers. For example, for a patient that needs a 1400 IU dose, PEG amounts were calculated using a 1000 IU vial plus 400 IU of a 500 IU vial, instead of using a 2000 IU vial, where factor would be wasted. All PEG molecules were treated equally, whether conjugated or present as an excipient or derivative, and regardless of size or structure.

Because to date there are no defined PEG-related AEs, we investigated whether there were any similar AEs that could be attributed to PEG within the product PIs, FDA website, general internet searches, and websites that report AEs. Safety outcomes were investigated by searching the medical literature (PIs, FDA documents, Google™, and factmed.com) for any report of clinical PEG-related AEs, and by attempting to identify any salient pattern of AEs listed in the PI or reported to the FDA or other reporting organization that might reflect PEG toxicity. After compiling extensive lists of the most common AEs, rare AEs, and post-marketing AEs, we conducted specific internet searches for the product/brand name along with symptoms and conditions pertaining to the liver, kidney, and CNS, followed by a search for all reported AEs on factmed.com, looking for specific symptoms/conditions pertaining to the liver, kidney, and CNS. Specifically, the search terms used in conjunction with the therapeutic identity were (using rurioctocog alfa pegol as an example): “Adynovate renal events”, “Adynovate nephropathy”, “Adynovate nephro”, “Adynovate liver events”, “Adynovate hepatic events”, “Adynovate hepat”, “Adynovate neurological events”, “Adynovate neuro”, “Adynovate central nervous system events”, “Adynovate CNS”, “Adynovate dysfunction”, “Adynovate post marketing adverse”, and “Adynovate enzyme”.

## 3. Results

### 3.1. PEG Exposure

**Figure 2:**
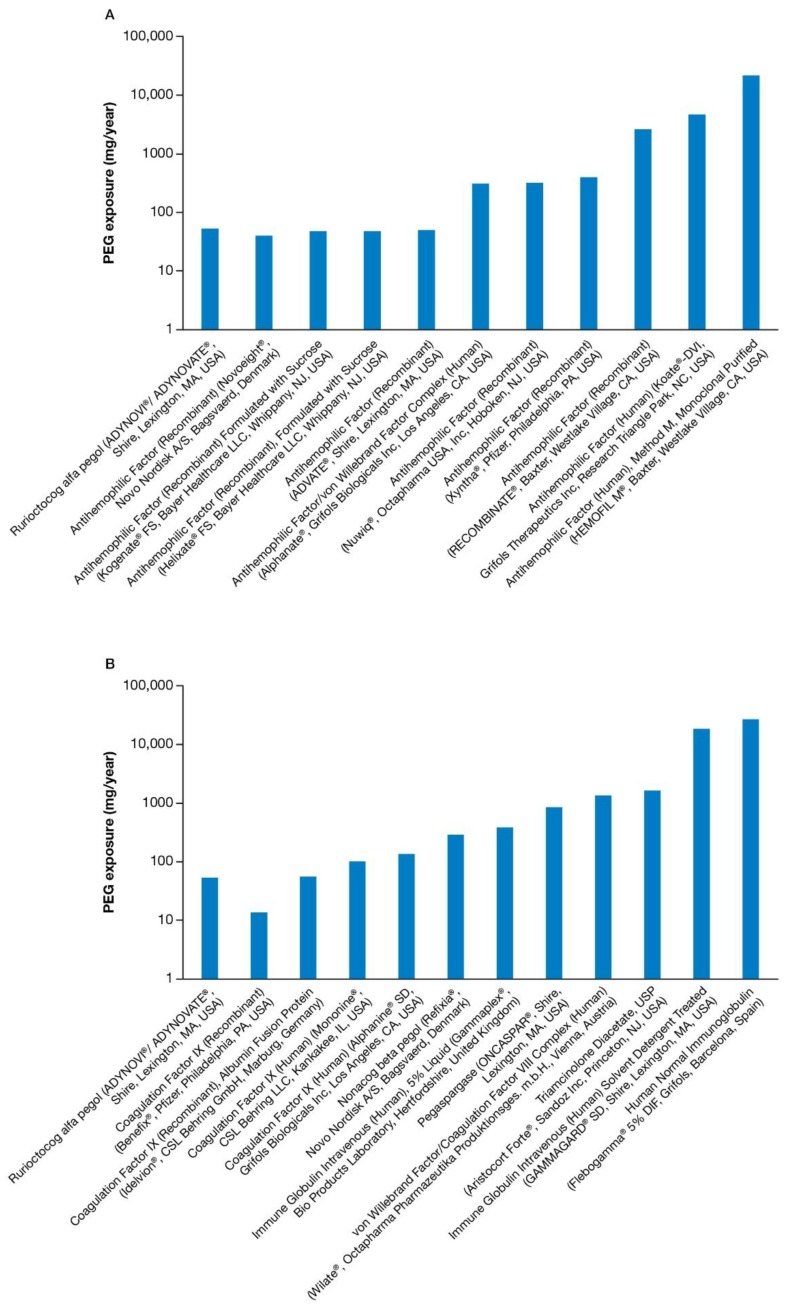
(**A**) Logarithmic plot of PEG exposure per year of rurioctocog alfa pegol versus FVIII prophylaxis in a 50 kg pediatric patient based on the highest dose analyzed. (**B**) Logarithmic plot of PEG exposure per year of rurioctocog alfa pegol versus FIX prophylaxis and other therapies in a 50 kg pediatric patient based on the highest dose analyzed.

In order to demonstrate a range of PEG exposure, Table 1 displays calculations using a theoretical 10 kg pediatric patient taking the minimum dose recommended by guidelines, while Figure 2A,B are based on calculations using a theoretical 50 kg pediatric patient taking the highest dose recommended by guidelines and/or the PI. A 10 kg pediatric patient would equate approximately to an 11-month-old child (estimated range of 7–23 months, based on weight-for-age data) and a 50 kg pediatric patient would equate approximately to a 14-year-old child (weight-for-age percentiles range 10+ years old) [24].

From our analyses of PEG-containing therapeutics, a patient’s exposure to conjugated PEG resulted in amounts much lower than that received from excipient or derivative PEG-containing therapies. For a child weighing 10 kg and receiving minimal prophylaxis dosages of FVIII, total PEG exposure ranged from <1–2730 mg/year; for prophylaxis with FIX products the range was <1–168 mg/year. For other therapies, total PEG exposure ranged from 2–2674 mg/year (Table 1). For a child weighing 50 kg and receiving maximum prophylaxis dosages of FVIII, total PEG exposure ranged from 40–21,840 mg/year; for prophylaxis with FIX products, the range was 13–1342 mg/year. For other therapies, total PEG exposure ranged from 383–27,743 mg/year (Figure 2A,B). The figures are displayed with a logarithmic axis due to the wide variability in the amounts of total PEG exposure within the three groups. Both figures show rurioctocog alfa pegol as a standard for comparison. PEG exposure for all the therapies in Figure 2A,B, and Table 1 represent the sum of conjugated, derivative, and excipient amounts, if present. Rurioctocog alfa pegol and nonacog beta pegol contain both conjugate and derivative forms of PEG, while pegaspargase contains only conjugated PEG. All other therapies contain excipient or derivative PEG only.

### 3.2. Adverse Events

There were no AEs consistently reported among the PIs for each therapy analyzed, whether listed as common, rare, or post-marketing reported AEs. Taking the preclinical toxicology findings into account, AEs involving the central nervous system (CNS), hepatic, and renal systems were analyzed. Factmed.com lists AEs reported to the FDA post launch. Due to the large number of AEs reported, especially pertaining to therapies on the market for decades, the preclinical findings were used as a search reference. There were no similarities within or between the FVIII or FIX product groups. In the other group, the only trend, which is stated as such in each PI, was amongst the human intravenous immunoglobulin and corticosteroid therapies. Within the immunoglobulin therapies, there are expected AEs that are attributed to this particular class of therapy including hypersensitivity, thrombosis, renal dysfunction, and failure. Other trends involved: flushing, headache, dizziness, chills, muscle cramps, back/joint pain, fever, nausea, and vomiting.

From our analysis, the administration of human immunoglobulin (Flebogamma^®^ 5% DIF, Grifols, Barcelona, Spain) exposes a 50 kg pediatric patient to over 27 g of derivative PEG per year, yet none of the AEs described in the literature could be attributable to the CNS, liver or kidneys. The most commonly reported AEs (affecting ≥5% of pediatric patients) are: headache, pyrexia, hypotension, tachycardia, diastolic hypotension, nausea, abdominal pain, diarrhea, pain, and vomiting (Supplemental Table S2) [29].

From our analysis, a 50 kg patient on the highest dose of pegaspargase receives the largest amount of conjugate PEG exposure (0.83 g per year). Pegaspargase is used to treat patients with acute lymphoblastic leukemia. Since these patients receive pegaspargase only for the duration of the episode unlike hemophilia A patients that require life-long prophylaxis, the 0.83 g of conjugate PEG can only be used in comparing PEG exposure over a year and not for lifetime use. Also, pegaspargase is typically used within specific protocols, and the total amount of pegaspargase may differ among different protocols and treatment arms within specific protocols. AEs affecting many systems (e.g., metabolic, cardiovascular, neurologic, hematologic, and digestive) were reported (Figure 2B; Supplemental Table S2). The most common AEs with pegaspargase (≥2%) are allergic reactions (including anaphylaxis), hyperglycemia, pancreatitis, CNS thrombosis, coagulopathy, hyperbilirubinemia, and elevated transaminases [30]. Without publicly available preclinical toxicity studies, it is hard to discern which of these manifestations, if any, may be PEG-related.

Table 2 demonstrates AE results, specific to the liver, kidney and CNS, for FVIII, FIX and other PEG-containing products. The supplementary tables list AEs, including those reported post-marketing and those listed in the PI for each therapy. There was no clear trend seen (Table 1, Supplementary Tables S1 and S2), except for immune globulin and corticosteroids where the PIs specifically state AEs that are attributable to these classes of therapies. There were no reports in the medical literature of clinical safety issues or signals specifically attributed to long-term exposure to PEG, and no recognizable patterns or trends of CNS, hepatic, or renal AEs.

## 4. Discussion

In this study, we presented our findings based on publicly available data regarding estimated annual PEG exposure in children with hemophilia receiving prophylactic factor replacement therapy, as well as in pediatric patients being treated with other IV or IM PEG-containing therapies. We showed that there is a wide range of PEG exposure, whether in a conjugated, derivative, or excipient form. Notably, estimated annual exposure to PEG from rurioctocog alfa pegol, including derivative and conjugated forms, is comparable to or lower than PEG exposure with other FVIII products [10,14].

In order to look for the clinical implications of long-term PEG exposure, it would be most useful if there were a generally accepted definition of a PEG-related AE. Unfortunately, there is no point of guidance since there are no definitive PEG-related AEs in humans. Defining a PEG-related AE is not as straightforward as it is with known toxic substances because no AEs have been specifically attributed to PEG. With respect to our analysis of AEs, a comprehensive search of data in the public domain did not reveal any AEs specifically attributed to PEG exposure, nor did we identify a pattern of events that may plausibly have been connected to PEG exposure. Despite paying particular attention to organ systems that appeared to be affected by ultra-high dose PEG exposure in preclinical toxicology studies (e.g., the CNS, kidney, liver), we were unable to detect a pattern of AEs suggestive of possible PEG toxicity.

Given this apparent lack of toxicity amongst pediatric patient populations receiving long-term treatment with therapies containing excipient, derivative, or conjugated PEG, it is most likely that the observed effects in animal models are attributable to the extremely large doses that were used. In theory, vacuolation could occur in humans that are being treated with prophylactic PEG-containing therapy, but because vacuolation cannot be meaningfully measured or monitored in humans due to the lack of appropriate biomarkers and the fact that relevant tissue biopsies are not feasible, the only way to investigate the effects of PEG exposure is to examine potentially related AEs. Our search did not reveal any patterns for related AEs. Our findings on safety, based on publicly available data, are consistent with others that have investigated PEG safety [2,3,4].

Preclinical in vivo toxicity studies with pediatric-indicated PEGylated drugs have shown certain conditions must be met for vacuolation within choroid plexus ependymal cells to be observed: the study used cynomolgus monkeys as the animal model, the PEG administered was >40 kDa, the length of the study was ≥6 weeks, and the dose provided exposure of ≥0.4 μmol/kg/month (approximately 0.8 g/month for a 50 kg human) [10,14].

Toxicology studies on small (≤10 kDa) PEG molecules have been performed via different administration routes, intravenous included. Monkeys treated intravenously with large doses of approximately 3 g/kg/day with 60% PEG 400 for up to one month had reduced appetite, infusion-site inflammation, and a greasy texture and edema of the lower extremities [3]. On the other hand, toxicology studies with PEGylated proteins have not revealed many PEGylated toxicities in animals. A twice-weekly subcutaneous administration of up to 150 μg/kg PEG-interferon in cynomolgus monkeys over a period of 13 weeks showed that it was well tolerated except for known interferon toxicity. Similar findings were seen with PEG-intron in repeated dose toxicity studies and clinical studies [3,23,31].

In preclinical toxicology in vivo studies on certolizumab pegol (Cimzia^®^, UCB, Inc., Smyrna, GA, USA) (which has a 40 kDa PEG moiety) with chronic high doses, there was an observation of organ-specific vacuolation in macrophages that resolved with the conclusion of dosing without permanent functional changes except at the highest weekly dose of 100 mg/kg in a 52-week study in cynomolgus monkeys (a dose that is an order of magnitude greater than the prescribing dose) [4]. Similar chronic high dosed toxicology studies with methoxy polyethylene glycol-epoetin beta (Mircera^®^, Vifor [International] Inc., St. Gallen, Switzerland) (30 kDa PEG) and peginterferon alfa-2a (PEGASYS^®^, Genentech USA, Inc., San Francisco, CA, USA) (40 kDa PEG), showed no PEG-related effects [4].

For hemophilia B, nonacog beta pegol was only recently approved in Europe and in the United States in 2017. We analyzed this therapy according to the European Medicines Agency Summary of Product Characteristics, since it was approved for prophylaxis [32]. Nonacog beta pegol is approved in Europe for the treatment and prophylaxis of bleeding in adults and children over 12 years with hemophilia B. The prophylactic dose is 40 IU/kg/week, which is significantly lower than the maximum doses used in the animal study [7,32]. The FDA approved nonacog beta pegol in adults and children for on-demand and perioperative treatment only, due to concerns observed in animals administered repeat doses that showed accumulation of PEG in the choroid plexus [7,22,30]. In general, there were no detectable trends of toxicity attributed to PEG accumulation from the use of the PEGylated products we analyzed, except for nonacog beta pegol. In the FDA-approved PI for nonacog beta pegol, there is a warning about the preclinical observation of PEG accumulation in the choroid plexus. After repeat-dosing in immune-deficient rats (40–1200 IU/kg/week for 26 weeks) and immune-competent monkeys (350–3750 IU/kg/week for four weeks), an accumulation of the 40 kDa PEG was found in the choroid plexus in a majority of the animals, but with no other associated morphological changes or atypical clinical signs [7].

The FDA-approved PI for nonacog beta pegol states that the potential clinical implications of these findings in animals are unknown and HCPs should practice caution with pediatric patients [32]. Also, there were no adverse neurologic effects of PEG reported in infants, children, and adolescents exposed to this therapy during clinical trials [22]. It must be noted that FVIII and FIX have different biodistribution patterns [33]. The difference in biodistribution is due to the FIX component in nonacog beta pegol, which is a much smaller molecular weight protein than the FVIII component in rurioctocog alfa pegol and the PEG conjugated is 40 kDa, which is twice the 20 kDa PEG used in rurioctocog alfa pegol. These differences in the therapeutic factor portion, factor sizes, and the PEG sizes, will affect how these therapies circulate through the body and how they are eliminated [6,7,10,34,35,36]. The FDA’s safety concerns on nonacog beta pegol are due to the accumulation of PEG in the choroid plexus in preclinical studies, which could be attributable to the large size difference and subsequent altered biodistribution. Because of this, comparisons between prophylactic use of rurioctocog alfa pegol and nonacog beta pegol may not be meaningful, since the biological portion of the therapy might influence the biodistribution and downstream effects, not the PEG portion of the therapy.

This study has several limitations. One limitation is the potential differences between excipient, derivative, and conjugated PEG. While we do not address metabolism and elimination of PEG here, the fact that PEG has no specific cellular receptors makes it somewhat more likely that the behavior of the molecule in humans would be similar to that observed in animal experiments. Due to the inert chemical nature of PEG, studying its metabolism in humans and other animals is challenging. PEG does not react with other molecules, and cannot be labelled or detected with established molecule labelling or detection reagents. Another consequence of the absent reactivity of PEG is that there are no biomarkers to easily detect it in tissues and organs. However, conjugated PEG follows the elimination pathway of the protein portion of the therapy, resulting in potentially differential exposure to PEG across various organs and tissues. Specifically, for rurioctocog alfa pegol, it has been shown that even at high doses, the conjugated PEG therapy is completely eliminated, reducing the risk of accumulation [10].

Another limitation of this study is the inherent difficulty in translating preclinical animal toxicology study results to clinical manifestations or AEs. Toxicology studies involving PEGylated proteins are usually performed before human testing occurs to provide safety information. With small organic molecules, toxicology studies are paired with metabolic studies to understand the biological fate of the molecule, but these are difficult studies to perform on PEGylated proteins [3]. Until we understand clearly if there is a correlation of PEG on the human system, we can only look for patterns amongst the publicly reported AEs. Finally, given the lack of any clearly defined adverse event specifically attributable to PEG exposure in humans, we cannot be completely confident in our finding that none have occurred. As always, “absence of evidence is not evidence of absence.”

In this article, we have investigated and addressed the concern among some HCPs about PEG exposure in the pediatric population. We showed that total PEG exposure varies widely depending on indicated doses and disease indication, and is mostly attributable to derivative PEG, while conjugate PEG amounts are low in comparison. There were no consistently reported AEs amongst the therapies we investigated, perhaps suggesting that PEG in these therapies does not lead to discernable clinical manifestations. Ongoing surveillance and clinical experience may provide additional evidence to further clarify the safety of the long-term treatment of children and adolescents with PEG-containing therapies.

## Figures and Tables

**Table 1:** Estimated PEG exposure in a 10 kg pediatric patient from the minimal dose indicated from FDA-approved IV or IM therapies with pediatric indications.

Product (Brand Name, Manufacturer)/Year Approved	Dosage	Peg Conjugated		Peg as Excipient		Type of Peg
		Per Dose (mg)	Per Year (mg)	Per Dose (mg)	Per Year (mg)	
FVIII Replacement Therapies						
Rurioctocog alfa pegol (ADYNOVI^®^/ADYNOVATE^®^, Shire, Lexington, MA, USA)/2015	50 IU/kg	<1	5	<1	17	20 kDa PEG *, PS80
Antihemophilic Factor (Recombinant) (ADVATE^®^, SHIRE, Lexington, MA, USA)/2003	25 IU/kg	0	0	<1	50	PS80
Antihemophilic Factor/von Willebrand Factor Complex (Human) (Alphanate^®^, Grifols Biologicals Inc., Los Angeles, CA, USA)/1978	25 IU/kg	0	0	<1	39	PS80/PEG mixture
Antihemophilic Factor (Recombinant), Formulated with Sucrose (Helixate FS^®^, Bayer Healthcare LLC, Whippany, NJ, USA)/1993	25 IU/kg	0	0	<1	24	PS80
Antihemophilic Factor (Human), Method M, Monoclonal Purified (HEMOFIL M^®^, Baxter, Westlake Village, CA, USA)/1966	25 IU/kg	0	0	18	2730	PEG 3350
Antihemophilic Factor (Human) (Koate^®^-DVI, Grifols Therapeutics Inc., Research Triangle Park, NC, USA)/1974	25 IU/kg	0	0	8	1183	PEG + PS80
Antihemophilic Factor (Recombinant) Formulated with Sucrose (Kogenate^®^ FS, Bayer Healthcare LLC, Whippany, NJ, USA)/1993	25 IU/kg	0	0	<1	24	PS80
Antihemophilic Factor (Recombinant) (NovoEight^®^, Novo Nordisk A/S, Bagsvaerd, Denmark)/2013	25 IU/kg	0	0	<1	40	PS80
Antihemophilic Factor (Recombinant) (Nuwiq^®^, Octapharma USA, INC, Hoboken, NJ, USA)/2015	25 IU/kg	0	0	2	323	P188
Antihemophilic Factor (Recombinant) (RECOMBINATE^®^, Baxter, Westlake Village, CA, USA)/1992	25 IU/kg	0	0	15	2378	PEG 3350 + PS80
Antihemophilic Factor (Recombinant) (Xyntha^®^, Pfizer, Philadelphia, PA, USA)/2008	25 IU/kg	0	0	3	402	PS80
von Willebrand Factor/Coagulation Factor VIII Complex (Human) (Wilate^®^, Octapharma Pharmazeutika Produktionsges m.b.H., Vienna, Austria)/2009	25 IU/kg	0	0	2	168	PS80
Factor IX (FIX) Replacement Therapies						
Nonacog beta pegol (Refixia^® †^, Novo Nordisk A/S, Bagsvaerd, Denmark)/2017	40 IU/kg	1	56	<1	5	40 kDa PEG *, PS80
Coagulation Factor IX (Human) (Alphanine^®^ SD, Grifols Biologicals Inc., Los Angeles, CA, USA)/1998	25 IU/kg	0	0	<1	17	PS80
Coagulation Factor IX (Recombinant) (Benefix^®^, Pfizer, Philadelphia, PA, USA)/1997	25 IU/kg	0	0	<1	13	PS80
Coagulation Factor IX (Recombinant), Albumin Fusion Protein (Idelvion^®^, CSL Behring GmbH, Marburg, Germany)/2016	25 IU/kg	0	0	<1	5	PS80
Coagulation Factor IX (Human) (Mononine^®^, CSL Behring LLC, Kankakee, IL, USA)/1992	25 IU/kg	0	0	<1	13	PS80
Other Pediatric-indicated Therapies						
Pegaspargase (ONCASPAR^®^, Shire, Lexington, MA, USA)/1994	2500 IU/m^2^	11	274	0	0	5 kDa PEG *
Triamcinolone Diacetate, USP (Aristocort Forte^®^, Sandoz Inc., Princeton, NJ, USA)/1961	40 mg/dose	0	0	31	1627	PEG + PS80
Human Normal Immunoglobulin (Flebogamma^®^ 5% DIF, Grifols, Barcelona, Spain)/2006	300 mg/kg	0	0	180	2674	PEG
Immune Globulin Intravenous (Human) Solvent Detergent Treated (GAMMAGARD^®^ SD, Shire, Lexington, MA, USA)/1986	300 g/kg	0	0	124	1840	PEG + PS80
Immune Globulin Intravenous (Human), 5% Liquid (Gammaplex^®^, Bio Products Laboratory, Hertfordshire, UK)/2009	300 mg/kg	0	0	2	29	PS80

Note: The “0” entry signifies the lack of that particular PEG in the therapy. * Conjugated PEG. ^†^ The nonacog beta pegol entry is calculated according to the EMA-approved prophylaxis indication, which is indicated for children >12 years old; therefore, the 10 kg may not be applicable, but is used here for consistency across all products.

**Table 2:** Liver, kidney and CNS safety information for the pediatric-indicated therapies sourced from prescribing information, FDA reports and factmed.com.

Drug	Renal Adverse Events	Liver Adverse Events	CNS Adverse Events
Rurioctocog alfa pegol (ADYNOVI^®^/ADYNOVATE^®^, Shire, Lexington, MA, USA)	NA	NA	NA
Antihemophilic Factor (Recombinant) (ADVATE^®^, Shire, Lexington, MA, USA)	NA	NA	NA
Antihemophilic Factor/von Willebrand Factor Complex (Human) (Alphanate^®^, Grifols Biologicals Inc., Los Angeles, CA, USA)	NA	NA	NA
Antihemophilic Factor (Recombinant), Formulated with Sucrose (Helixate FS^®^, Bayer Healthcare LLC, Whippany, NJ, USA)	NA	NA	NA
Antihemophilic Factor (Human), Method M, Monoclonal Purified (HEMOFIL M^®^, Baxter, Westlake Village, CA, USA)	NA	NA	NA
Antihemophilic Factor (Human) (Koate^®^-DVI, Grifols Therapeutics Inc., Research Triangle Park, NC, USA)	NA	NA	NA
Antihemophilic Factor (Recombinant) Formulated with Sucrose (Kogenate^®^ FS, Bayer Healthcare LLC, Whippany, NJ, USA)	NA	NA	NA
Antihemophilic Factor (Recombinant) (NovoEight^®^, Novo Nordisk A/S, Bagsvaerd, Denmark)	NA	Increased hepatic enzymes	NA
Antihemophilic Factor (Recombinant) (Nuwiq^®^, Octapharma USA, Inc., Hoboken, NJ, USA)	NA	NA	NA
Antihemophilic Factor (Recombinant) (RECOMBINATE^®^, Baxter, Westlake Village, CA, USA)	NA	NA	NA
Antihemophilic Factor (Recombinant) (Xyntha^®^, Pfizer, Philadelphia, PA, USA)	NA	NA	NA
Coagulation Factor IX (Human) (AlphaNine^®^ SD, Grifols Biologicals Inc., Los Angeles, CA, USA)	NA	NA	NA
Coagulation Factor IX (Recombinant), Albumin Fusion Protein (Idelvion^®^, CSL Behring GMBH, Marburg, Germany)	NA	NA	Dizziness
Nonacog beta pegol (Refixia^®^/Rebinyn^®^, Novo Nordisk A/S, Bagsvaerd, Denmark)	NA	NA	NA
von Willebrand Factor/Coagulation Factor VIII Complex (Human) (Wilate^®^, Octapharma Pharmazeutika Produktionsges m.b.H., Vienna, Austria)	NA	NA	NA
Coagulation Factor IX (Recombinant) (Benefix^®^, Pfizer, Philadelphia, PA, USA)	Rare: Renal infarct	NA	NA
Coagulation Factor IX (Human) (Mononine^®^, CSL Behring LLC, Kankakee, IL, USA)	Nephrotic syndrome in hemophilia patients with a history of hypersensitivity reactions	NA	NA
Immune Globulin Intravenous (Human) Solvent Detergent Treated (Gammagard^®^ SD, Shire, Lexington, MA, USA)	Common among IG products: Acute renal dysfunction/failure, osmotic nephropathy	Common amongst IG products: hepatic dysfunction	Common among IG products: Coma, loss of consciousness, seizures, tremor, aseptic meningitis syndrome
Immune Globulin Intravenous (Human), 5% Liquid (Gammaplex^®^, Bio Products Laboratory, Hertfordshire, UK)	Common among IG products: Acute renal dysfunction/failure, osmotic nephropathy	Common among IG products: hepatic dysfunction, abdominal pain	Migraine, aseptic meningitis Common among IG products: Coma, loss of consciousness, seizures, tremor, aseptic meningitis syndrome
Human Normal Immunoglobulin (Flebogamma^®^ 5% DIF, Grifols, Barcelona, Spain)	Common among IG products: Acute renal dysfunction/failure, osmotic nephropathy	Common among IG products: Hepatic dysfunction, abdominal pain	Common among IG products: Coma, loss of consciousness, seizures, tremor, aseptic meningitis syndrome
Triamcinolone Diacetate, USP (Aristocort Forte^®^, Sandoz Inc., Princeton, NJ, USA)	Common among corticosteroids: elevation of blood pressure, salt and water retention, and increased excretion of potassium and calcium, renal failure	Bowel/bladder dysfunction, elevation in serum liver enzyme levels, hepatomegaly Common among corticosteroids: hepatic failure	Convulsions, depression, emotional instability, euphoria, headache, increased intracranial pressure with papilledema, insomnia, mood swings, neuritis, neuropathy, paresthesia, personality changes, psychic disorders, vertigo. Arachnoiditis, meningitis, paraparesis/paraplegia, and sensory disturbances Common neurological AEs among corticosteroids: Spinal cord infarction, paraplegia, quadriplegia, cortical blindness, seizure, neurological deterioration, and stroke
Pegaspargase (ONCASPAR^®^, Shire, Lexington, MA, USA)	Increased BUN, increased creatinine, urinary frequency, hematuria due to thrombocytopenia, severe hemorrhagic cystitis, renal dysfunction, and renal failure.	Jaundice, ascites, and hypoalbuminemia, hepatomegaly, fatty changes in the liver and liver failure. Hepatotoxicity and abnormal liver function, including elevations of AST, ALT, ALP, bilirubin, and depression of SA, and plasma fibrinogen	CNS thrombosis/hemorrhage

ALT, alanine aminotransferase; ALP, alkaline phosphatase; AST, aspartate aminotransferase; BUN, blood urea nitrogen; IG, Immunoglobulin; SA, serum albumin.

## Supplementary Materials

The following are available online at http://www.mdpi.com/1424-8247/11/3/75/s1, Table S1: Factor VIII and IX Therapy Adverse Events, Table S2: Other Therapy Adverse Events.
