# Supplementary material for: Polyethylene Glycol Exposure with Antihemophilic Factor (Recombinant), PEGylated (rurioctocog alfa pegol) and Other Therapies Indicated for the Pediatric Population: History and Safety
Source: Pharmaceuticals (Basel). 2018 Jul 26;11(3):75. doi: 10.3390/ph11030075 (PMC6160981; doi:10.3390/ph11030075)
Supplement: Supplementary file 1 [file pharmaceuticals-11-00075-s001.zip › pharmaceuticals-327060-supplementary.pdf]

pharmaceuticals

MDPI

Polyethylene Glycol Exposure with Antihemophilic Factor (Recombinant), PEGylated (rurioctocog alfa pegol) and Other Therapies Indicated for the Pediatric Population: History and Safety

Reinhard Stidl [1], Michael Denne [2], Jimena Goldstine [2], Bill Kadish [3], Katherine I. Korakas [3] and Peter L. Turecek *

Supplemental Table S1. Factor VIII and IX Therapy Adverse Events

**Drug Name**	**Adverse Events Listed in PI**	**Post-Marketing-reported Adverse Events**
**Factor VIII Therapies**		
Rurioctocog alfa pegol (Shire)	Headache, nausea, diarrhea, hypersensitivity, headache, rash, flushing	NA
Antihemophilic factor (recombinant) (Shire)	Pyrexia, headache, cough, nasopharyngitis, vomiting, arthralgia, limb injury, upper respiratory tract infection, pharyngolaryngeal pain, nasal congestion, diarrhea, nausea, pain, rash, ear infection, procedural pain, rhinorrhea	Anaphylaxis, immune system hypersensitivity, FVIII inhibition, injection-site reaction, chills, fatigue/malaise, chest discomfort/pain, less-than-expected therapeutic effect
Antihemophilic factor/von Willebrand factor complex [human] (Grifols)	Pruritus, headache, back pain, paresthesia, respiratory distress, facial edema, pain, rash, chills, pulmonary embolism, hemorrhage, hematocrit decrease, orthostatic hypotension	Paresthesia, respiratory distress, facial edema, pain, rash, and chills
Antihemophilic actor (recombinant), formulated with Sucrose (Bayer)	Inhibitor formation, skin-associated hypersensitivity reactions, infusion site reactions, central venous access device associated infections, systemic hypersensitivity reactions (bronchospastic reactions, hypotension and anaphylaxis)	Dysgeusia

Antihemophilic factor (human), Method M, monoclonal purified (Baxter)	Factor VIII inhibition, dizziness, headache, dysgeusia, pyrexia, infusion site inflammation, chest tightness, fuzziness	Anaphylactic reaction, hypersensitivity, visual impairment, ocular hyperemia, cyanosis, bradycardia, tachycardia, hypotension, flushing, bronchospasm, dyspnea, cough, hyperventilation, diarrhea, vomiting, nausea, abdominal pain, urticaria, rash, pruritus, hyperhidrosis, inhibitor development, musculoskeletal pain, facial edema, edema, chills, fatigue, chest pain, irritability
Antihemophilic factor (human) (Grifols)	Allergic-type reactions, paresthesia, vision blurred, headache, nausea, abdominal pain, and feeling jittery	
Antihemophilic factor (recombinant) formulated with sucrose (Bayer)	Inhibitor formation, skin-associated hypersensitivity reactions, infusion site reactions, central venous access device associated infections, pyrexia	Dysgeusia
Antihemophilic factor (recombinant) (Novo Nordisk)	Injection-site reactions, increased hepatic enzymes, and pyrexia	
Antihemophilic factor (recombinant) (Octapharma)	Paresthesia, headache, injection-site inflammation, injection-site pain, non-neutralizing anti-factor VIII antibody formation, back pain, vertigo, and dry mouth	NA
Antihemophilic factor (recombinant) (Baxter)	Chills, flushing, rash, epistaxis, nausea, fatigue, pyrexia, ear infections, acoustic stimulation tests abnormal, pain in extremity, dizziness, tremors, pharyngolaryngeal pain, hyperhidrosis, pruritus, epistaxis, hematoma, hypotension, pallor, peripheral coldness	Factor VIII inhibition, tachycardia, cyanosis, vomiting, abdominal pain, malaise, injection-site reactions, chest pain, chest discomfort, anaphylactic reaction, hypersensitivity, loss of consciousness, headache, paresthesia, dyspnea, cough, laryngeal edema, angioedema, urticaria, erythema
Antihemophilic factor (recombinant) (Pfizer)	Headache, arthralgia, pyrexia, inhibitor development, cough, vomiting, diarrhea, asthenia, nausea	Anaphylaxis, Inadequate therapeutic response
**Factor IX Therapies**		

Coagulation factor IX (human) (Grifols)	Allergic reactions, mild chills, nausea or stinging at the infusion site, thrombosis or disseminated intravascular coagulation	NA
von Willebrand Factor/coagulation factor VIII complex (human) (Octapharma)	Hypersensitivity reactions, urticaria, and dizziness	Dyspnea, nausea, vomiting, rash, headache, tachycardia, flushing, hypotension, chills, cough, chest discomfort, abdominal pain, pyrexia, factor VIII inhibition, anaphylactic reaction and paresthesia
Coagulation factor IX (Recombinant) (Pfizer)	Nausea, injection-site reaction and pain, headache, dizziness, rash, systemic hypersensitivity reactions	Inadequate factor IX recovery, inadequate therapeutic response, inhibitor development, anaphylaxis, angioedema, dyspnea, hypotension, and thrombosis. DVT and peripheral thrombophlebitis reported
Nonacog beta pegol (Novo Nordisk)	Hypersensitivity, anaphylaxis, inhibitors, palpitations, nausea, pruritus, fatigue, hot flush, injection-site reactions	NA
Coagulation factor IX (human) (CSL Behring)	Headache, fever, chills, flushing, nausea, vomiting, tingling, lethargy, hives, stinging or burning at the infusion site or manifestations of allergic reactions. Some experienced ALT elevations	Anaphylaxis, angioedema, cyanosis, dyspnea, hypotension, thrombosis, inadequate therapeutic response, and inhibitor development
Coagulation factor IX (recombinant), albumin fusion protein (CSL Behring)	Headache, dizziness, hypersensitivity, rash, eczema	NA

The AEs were sourced from the prescribing information (PI) for each therapy and represent the most common and less common AEs observed during clinical trials. "Post-marketing AEs" are AEs listed in the PI as such.

pharmaceuticals

MDPI

Supplemental Table S2. Other Therapy Adverse Events

Drug	Most Common Adverse Events	Post-marketing Reported Adverse Events
Immune globulin intravenous (human) solvent detergent treated (Shire)	Headache, nausea, chills, fatigue, pyrexia, upper abdominal pain, diarrhea, back pain, infusion site pain, hyperhidrosis and flushing. Aseptic meningitis syndrome, anemia, hemolysis, lymphadenopathy, thrombocytopenia, anaphylactic shock, anaphylactic/anaphylactoid reaction, hypersensitivity, restlessness, cerebrovascular accident, transient ischemic attack, convulsion, dizziness, migraine, paresthesia, syncope, tremor, retinal vein thrombosis, eye pain, photophobia, visual disturbance, myocardial infarction, cyanosis, tachycardia, bradycardia, vena cava thrombosis, arterial thrombosis, deep vein thrombosis, hypotension, hypertension, pallor, thrombophlebitis, pulmonary embolism, pulmonary edema, bronchospasm, wheezing, cough, hyperventilation, hypoxia, throat tightness, abdominal pain, dyspepsia, hepatitis, angioedema, dermatitis, erythema, rash, arthralgia, myalgia, infusion site reaction, asthenia, edema, rigors, positive direct coombs Test.	Renal failure, thrombotic events, anaphylactic shock, aseptic meningitis and hemolysis
Immune globulin intravenous [human], 5% liquid (Bio Products Laboratory)	Headache, pyrexia, nasal congestion/edema, fatigue, nausea, hypertension, rash, hypotension, infusion site reaction, vomiting, myalgia, chills, tachycardia, chest pain/discomfort, pain, dizziness, malaise, dysuria, and dry skin, pruritus, dehydration, and arthralgia. Abdominal pain upper, gastritis, contusion, arthralgia, cough, anemia, ecchymosis, pruritus, dehydration, hypertension, neck pain, sinusitis, rhinitis, vertigo, asthenia, cystitis, UTI, eczema, bronchitis	Dizziness, pulmonary embolism, dyspnea, respiratory distress, myocardial infarction, other thromboembolic event, migraine, aseptic meningitis, rash, urticarial, musculoskeletal pain
Human normal immunoglobulin (Grifols)	Headache, pyrexia/fever, pain, infusion site reactions, diarrhea, rigors or chills, urticaria, hypotension, tachycardia, nausea, abdominal pain, and vomiting. Hypertension, sinusitis, positive Coombs test, arthralgia, myalgia, dizziness, bronchitis, and hypotension	Hypersensitivity, headache, diarrhea, tachycardia, fever, fatigue, dizziness, malaise, chills, flushing, urticaria or other skin reactions, wheezing or other chest discomfort, nausea, vomiting, rigors, back pain, myalgia, arthralgia, and changes in blood pressure, acute renal dysfunction/failure, osmotic nephropathy, apnea, acute respiratory distress syndrome, transfusion-related acute lung, injury, cyanosis, hypoxemia, pulmonary edema, dyspnea, bronchospasm, Cardiac arrest, thromboembolism, vascular collapse, hypotension, Coma, loss of consciousness, seizures, tremor, aseptic meningitis syndrome, Stevens-Johnson syndrome, epidermolysis, erythema multiforme, dermatitis, pancytopenia, leukopenia, hemolysis, positive direct antiglobulin test, back pain, hepatic dysfunction, abdominal pain, pyrexia, rigors

Triamcinolone Diacetate, USP (Sandoz)	Allergic or hypersensitivity reactions, anaphylactoid reactions, anaphylaxis, angioedema. Bradycardia, cardiac arrest, cardiac arrhythmias, cardiac enlargement, circulatory collapse, congestive heart failure, fat embolism, hypertension, hypertrophic cardiomyopathy in premature infants, myocardial rupture following recent myocardial infarction, pulmonary edema, syncope, tachycardia, thromboembolism, thrombophlebitis, vasculitis. Acne, allergic dermatitis, cutaneous and subcutaneous atrophy, dry scaly skin, ecchymoses and petechiae, edema, erythema, hyperpigmentation, hypopigmentation, impaired wound healing, increased sweating, rash, sterile abscess, striae, suppressed reactions to skin tests, thin fragile skin, thinning scalp hair, urticaria. Decreased carbohydrate and glucose tolerance, development of cushingoid state, glycosuria, hirsutism, hypertrichosis, increased requirements for insulin or oral hypoglycemic agents in diabetes, manifestations of latent diabetes mellitus, menstrual irregularities, secondary adrenocortical and pituitary unresponsiveness, suppression of growth in pediatric patients. Congestive heart failure, fluid retention, hypokalemic alkalosis, potassium loss, sodium retention. Abdominal distention, increased appetite, nausea, pancreatitis, peptic ulcer with possible perforation and hemorrhage, perforation of the small and large intestine, ulcerative esophagitis. Negative nitrogen balance due to protein catabolism. Aseptic necrosis of femoral and humeral heads, calcinosis, Charcot-like arthropathy, loss of muscle mass, muscle weakness, osteoporosis, pathologic fracture of long bones, postinjection flare, steroid myopathy, tendon rupture, vertebral compression fractures. Convulsions, depression, emotional instability, euphoria, headache, increased intracranial pressure with papilledema, insomnia, mood swings, neuritis, neuropathy, paresthesia, personality changes, psychic disorders, vertigo. Arachnoiditis, meningitis, paraparesis/paraplegia, and sensory disturbances. Exophthalmos, glaucoma, increased intraocular pressure, posterior subcapsular cataracts, rare instances of blindness associated with periocular injections. Abnormal fat deposits, decreased resistance to infection, hiccups, increased or decreased motility and number of spermatozoa, injection-site infections following non-sterile administration, malaise, moon face, weight gain	NA
Pegaspargase (Shire)	Allergic reactions (including anaphylaxis), central nervous system (CNS) thrombosis, coagulopathy, elevated transaminases, hyperbilirubinemia, hyperglycemia, and pancreatitis	NA

The AEs were sourced from the PI for each therapy and represent the most common and less common AEs observed during clinical trials. "Post-marketing AEs" are AEs listed in the PI as such.
